# Aqueous Triple-Phase System in Microwell Array for Generating Uniform-Sized DNA Hydrogel Particles

**DOI:** 10.3389/fgene.2021.705022

**Published:** 2021-07-23

**Authors:** Marcos Kunihiro Masukawa, Yukiko Okuda, Masahiro Takinoue

**Affiliations:** Department of Computer Science, Tokyo Institute of Technology, Yokohama, Japan

**Keywords:** monodisperse, DNA hydrogel, microwell array, self-assembly, aqueous two-phase system, DNA nanotechnology, microfluidics, artificial cells

## Abstract

DNA hydrogels are notable for their biocompatibility and ability to incorporate DNA information and computing properties into self-assembled micrometric structures. These hydrogels are assembled by the thermal gelation of DNA motifs, a process which requires a high salt concentration and yields polydisperse hydrogel particles, thereby limiting their application and physicochemical characterization. In this study, we demonstrate that single, uniform DNA hydrogel particles can form inside aqueous/aqueous two-phase systems (ATPSs) assembled in a microwell array. In this process, uniform dextran droplets are formed in a microwell array inside a microfluidic device. The dextran droplets, which contain DNA motifs, are isolated from each other by an immiscible PEG solution containing magnesium ions and spermine, which enables the DNA hydrogel to undergo gelation. Upon thermal annealing of the device, we observed the formation of an aqueous triple-phase system in which uniform DNA hydrogel particles (the innermost aqueous phase) resided at the interface of the aqueous two-phase system of dextran and PEG. We expect ATPS microdroplet arrays to be used to manufacture other hydrogel microparticles and DNA/dextran/PEG aqueous triple-phase systems to serve as a highly parallel model for artificial cells and membraneless organelles.

## Introduction

Artificial cells and artificial membraneless organelles try to mimic molecular biology systems to develop an improved understanding of their biological counterparts, the origin of life (Szostak et al., 2001), by exploring the physicochemical essence of life systems (Noireaux et al., 2011; Takinoue and Takeuchi, 2011), and producing new smart and active soft materials (Swi Chang, 2005; Hagiya et al., 2014). Two important aspects of artificial mimics are selective uptake (Martin et al., 2016) and molecular crowding (Myhrvold et al., 2013; Tan et al., 2013), which are responsible for creating and maintaining systems that are out of equilibrium, such as those occurring in living beings. One of the models of artificial cells and membraneless organelles that can mimic these aspects is composed of aqueous/aqueous two-phase systems (ATPSs) created by liquid-liquid phase separation (LLPS) (Douliez et al., 2018). These systems, created by mixing immiscible polymer solutions or a polymer solution with a salt (Albertsson, 1971), can create emulsions with concentration gradients of the polymer (Iqbal et al., 2016; Zhao et al., 2016). One of the most studied ATPSs consists of dextran and polyethylene glycol (PEG), and was discovered over 60 years ago (Wesslén et al., 1959). Dextran and PEG ATPS can be used as templates to synthesize particles (Stenekes et al., 1999), concentrate substrates and enzymes, accelerate chemical reactions (Strulson et al., 2012) and enable the recovery of enzymes (Pollak and Whitesides, 1976), purify proteins (Fried and Chun, 1971; Schmidt et al., 1994), separate DNA fragments (Lis, 1980), and concentrate genomic DNA (Tsumoto et al., 2015; Nakatani et al., 2018). Dextran and PEG emulsions can be easily prepared by mixing and agitating polymer solutions, and consist of a continuous phase and a droplet phase. Nonetheless, emulsification by agitation produces polydisperse droplets. Polydisperse sizes are not desirable when the droplets are used as quantifiable models of cells or cellular components. Therefore, attempts have been made to use microfluidic devices to generate monodisperse dextran and PEG emulsions (Ziemecka et al., 2011; Cheung Shum et al., 2012; Song and Shum, 2012; Moon et al., 2015; Zhou et al., 2017), which have been used to encapsulate cells (Navi et al., 2018; Mastiani et al., 2019). However, they are inherently unstable and therefore cannot be used for further experiments without coalescing, requiring a stabilizing agent such as lysozyme fibrillosomes (Song et al., 2016), mineral nanoplates (Vis et al., 2015), or cellulose nanorods (Peddireddy et al., 2016). In this study, we demonstrate that a dextran-in-PEG (Dex/PEG) monodisperse emulsion^1^ without stabilizing agents can be created in a microwell array and that this stable emulsion can be used for the self-assembly of monodisperse DNA hydrogels.

DNA hydrogels are a class of soft materials composed in part or entirely of DNA (Um et al., 2006; Dong et al., 2020). These hydrogels allow conjugation with other DNA sequences and biological molecules to incorporate sensing (Cheng et al., 2009; Li et al., 2020), information processing (Yin et al., 2012), and actuating capabilities (Bi et al., 2020) into the structure. Additionally, the macroscopic properties of the material, such as the thermal sensitivity (Xing et al., 2011), pore size (Um et al., 2006), and viscosity (Fernandez-Castanon et al., 2018) can be tuned by the DNA sequences, which allows easy modification of the macroscopic properties of the material. Consequently, it is an ideal medium for gene editing (English et al., 2019), drug delivery systems (Mo et al., 2020), and sensors (Merindol et al., 2019). Similar to other hydrogels, their size affects the characterization of particles and might affect the delivery route or release of therapeutic agents (Mo et al., 2020), leading to the need for monodisperse DNA hydrogel particles.

**FIGURE 1:**
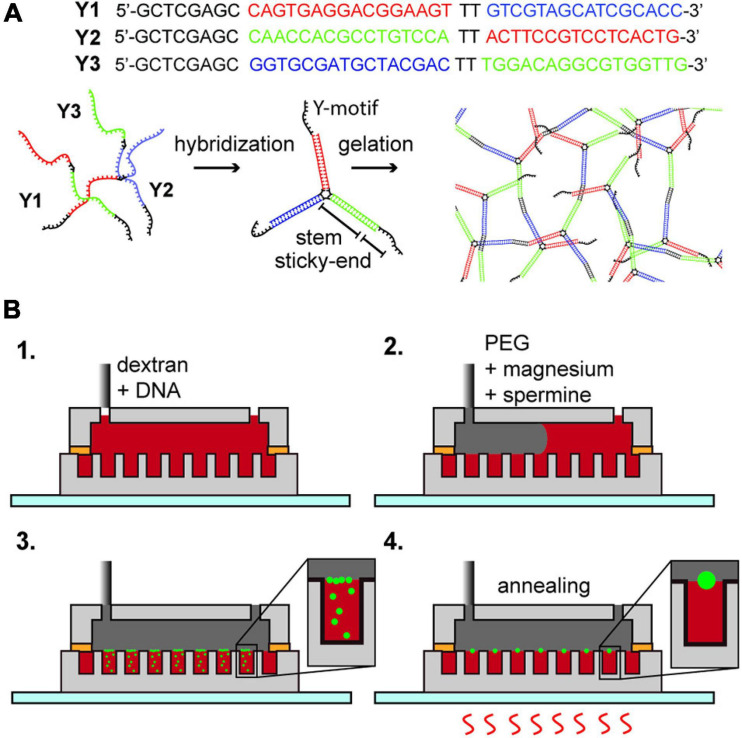
**(A)** DNA hydrogel; DNA Y1, Y2, and Y3 hybridize into a motif which forms a network structure by the hybridization of the sticky-end portion. **(B)** Formation of DNA hydrogel inside an aqueous two-phase system droplet array. (1) Insertion of dextran with Y1, Y2, and Y3 oligonucleotides. (2) Insertion of PEG containing magnesium ions and spermine. (3) Diffusion of magnesium ions and spermine into the dextran droplets, forming DNA aggregates at the interface. (4) Multiple thermal annealing of the device and generation of an aqueous triple-phase system DNA/dextran/PEG.

Branched DNA motifs, also known as DNA nanostars, form a special class of DNA hydrogels of which the size is challenging to control. In these structures, a set of oligonucleotides forms a junction with double-stranded branches, also referred to as arms or stems, terminated by a single-stranded region termed the sticky end (Figure 1A). The sticky ends interact, promoting the formation of a network of motifs (Sato et al., 2020). The viscosity of DNA hydrogels formed by branched motifs is controllable by the strength of interaction of the sticky ends and the temperature, allowing the reversible transition of these droplets from gel to liquid. DNA hydrogels in the liquid state, also known as DNA droplets, display distinct properties, such as the sequence-based incorporation of proteins/enzymatic fission (Sato et al., 2020), size control by enzymatic activity (Saleh et al., 2020), and the sequence-based control of adhesion/emulsification (Jeon et al., 2020).

Few alternatives are available to assemble monodisperse DNA hydrogels and DNA droplets owing to their mechanism of formation by nucleation and growth. Controlled heating and cooling can influence the size of the DNA hydrogel particles, but because of the random aspect of nucleation of particles and their coalescence, their size is polydisperse. Thus far, size control of hydrogels has involved compartmentalization using microfluidic devices and photolithographic techniques. Microfluidic devices have been used to produce monodisperse coacervates containing DNA inside a vesicle (Deng and Huck, 2017) and DNA hydrogels in water-in-oil (W/O) droplets (Kim et al., 2016). The disadvantage of these methods is that they require an oil phase, which must be removed during particle recovery. Photolithographic techniques rely on light-sensitive materials to induce the gelation of DNA hydrogels composed of branched DNA motifs (Kasahara et al., 2020); however, this method is not ideal for producing particles on the micrometer scale and can be incompatible with *in vivo* use.

In this study, we explored the size control of DNA hydrogels using ATPS. We produced a microarray of dextran droplets containing DNA surrounded by a continuous PEG phase containing magnesium ions and spermine, which induced the formation of DNA aggregates at the interface of each dextran droplet. By heating the device, DNA droplets formed inside the dextran phase, creating an aqueous triple-phase system. Cooling the device resulted in uniform-sized DNA hydrogel particles at the interface of the Dex/PEG droplets.

## Materials and Methods

### DNA Hydrogel Formation

We purchased the DNA strands named Y1, Y2, and Y3 (Eurofins genomics, sequence displayed in Figure 1A) as custom synthesized oligonucleotides by oligonucleotide purification cartridge (OPC) dissolved in pure water (Milli-Q water, 18.2 M Ω⋅cm resistivity at 25°C) at 100 μM concentration, used without further purification, and stored at −20°C until use. By the annealing of the mixture of Y1, Y2, and Y3 in a buffer solution, a branched DNA motif named Y-motif is first formed, and finally DNA hydrogels are formed (Figure 1A). We tested the formation of DNA hydrogels under three conditions, that is, in a dextran single-phase system in a bulk solution, a Dex/PEG two-phase system in a bulk solution, or a Dex/PEG two-phase system in a microwell array.

We prepared four different solutions (Supplementary Table 1). Solution (i) was composed of 10 mM Tris–HCl (pH 8.0) (Invitrogen by Life Technologies Japan, 15568-O25, LOT 2018-06-30), 8.33% w/w of dextran (molecular weight ∼200,000; Wako, 041-22612, LOT: WDE0888), 0.01% w/w of fluorophore-modified dextran (Tretramethylrhodamine isothiocyanate-modified dextran; Sigma-Aldrich, T1287-50MG, LOT:011M1861V; henceforth called rhodamine-dextran; excitation peak wavelength: 544 nm, emission peak wavelength: 570 nm), 8 μM of each of DNA strands Y1, Y2, and Y3, 2.5 mM of magnesium acetate tetrahydrate (Wako, 133-10012, LOT: KLN3877), 2.5 μM of spermine (Wako, 198-09811 LOT: STE3053) to favor DNA aggregation, and 2/10000 dilution of Quant-iT Oligreen ssDNA reagent (hereafter, Oligreen) (Thermo Fisher Scientific, 07582, LOT:1103056) to stain the DNA. Solution (ii) was almost the same as solution (i) but did not contain magnesium acetate or spermine. Solution (iii) contained 10 mM Tris–HCl (pH 8.0), 3.33 mM magnesium acetate, 1.66 μM spermine, and 8.33% w/w PEG (molecular weight ∼6,000; Wako, 169-09125, LOT: PTL1562). Solution (iv) was almost the same as solution (iii) but with 2.5 mM magnesium ions and 1.25 μM spermine.

The dextran single-phase system in a bulk solution consisted of 20 μL of solution (i) (Supplementary Table 2). The Dex/PEG two-phase system in a bulk solution was made by mixing and agitating 5 μL of solution (ii) with 15 μL of solution (iii), resulting in a Dex/PEG emulsion with 2.5 mM magnesium acetate and 1.25 μM spermine as a final concentration (Supplementary Table 2). For the gelation of DNA hydrogel by both systems, we loaded the samples in a PCR tube, heated the solution to 75°C for 1 min in a dry block heater (Nissin Thermo-block ND-M01), brought to room temperature for 3 min, and then heated again to 75°C for 1 min.

For the Dex/PEG two-phase system in a microwell array experiment (Figure 1B and Supplementary Table 2), we injected 50 μL of solution (ii) inside the microfluidic device; to remove air from inside the microwells, we placed the device in a vacuum and reinjected the solution multiple times until all the microwells were filled, and then removed the excess solution (ii) and slowly injected 100 μL of solution (iv) to make a Dex/PEG two-phase system in the microwell array. Because the volume of the device chamber is much larger than the volume of solution (ii) inside the microwells, the concentrations of magnesium ions and spermine were approximately 2.5 mM and 1.25 μM, respectively. The microwell array device was then annealed on a hotplate (As One, Ninos ND-2) by heating at 75°C for 1 min, letting it cool to room temperature for 3 min (first annealing round), and heated at 75°C for 1 min (second annealing round). We tested the formation of hydrogels in two-phase systems in microwells sized 50 and 100 μm in diameter with either 1, 4, or 8 μM of each DNA strand in the dextran solution, depending on the experiment.

### Microfluidic Device Fabrication

The microfluidic device (Figure 1B and Supplementary Figure 1) was composed of a polydimethylsiloxane (PDMS) elastomer. The bottom part had an array of cylindrical microwells (50, 100, or 200 μm in diameter; ∼100 μm in depth), and the upper part had a flow channel (5 mm width, ∼100 μm height). The bottom part was made by casting a PDMS mix [silicone elastomer mixed with its curing agent in a ratio of 1:10 (SILPOT 184, Dow Corning Toray; LOT: 0008494274 and H05218V004, respectively)] on a photoresist mold on a silicon wafer. To prepare the photoresist mold, we dried a 2-inch silicon wafer (MCO, GA2002) for 15 min at 120°C and then cooled it down to room temperature. We spin-coated 1 mL of photoresist SU-3050 (Kayaku Advanced Materials (MicroChem), LOT: 16110795) on a silicon wafer with a maximum rotational frequency of 3000 rpm for 30 s, yielding a photoresist layer with a thickness of ∼50 μm. The mold was baked for 15 min at 95°C, let it cool down, and the spin coating was repeated until the thickness of the photoresist was approximately 100 μm. We exposed the photoresist with a maskless pattern generator μPG-101 (Heidelberg Instruments) with 3 μm resolution, 100% of 10 mW power, and four consecutive exposures per line. For the exposure, we designed a pattern of pillars in a square lattice with diameters of 50, 100, or 200 μm. The exposed photoresist mold was baked at 95°C for 3 min and then washed in a 1-methoxy-2-propyl acetate-based SU-8 developer (Kayaku Advanced Materials (MicroChem), LOT:17010033). We removed the remaining unexposed photoresist between the pillars by placing the developer solution with the mold in an ultrasound bath. The mold was washed with 99.5% isopropyl alcohol (IPA) and the development process was repeated. The device was post-baked for 15 min at 150 °C to improve the adherence of the photoresist to the silicon wafer. Following post-baking, we placed the photoresist mold on a ϕ90-mm polypropylene Petri dish set together with 5 mm thick glass spacers around the wafer to cast the microwell array. We poured approximately 3 mL of the PDMS mix on the silicon mold and degassed it by placing it in a vacuum. Next, we pressed a ϕ87-mm dish upside down on the top of the silicon wafer, sandwiching the wafer between the two dishes. We curated this set at 95°C for approximately 15 min, carefully removed the PDMS microwell array, and placed it with the microwells upward on a glass slide.

For the flow chamber, we cut a sticker sheet (Tack seal, University Co-op., A4, IJRT-AA4N, thickness ∼100 μm) with a cutter plotter (Graphtec CE7000-40), shaped like a hexagon with sides 5, 5, 10, 5, 5, and 10 mm. The cut sticker was then stuck on a glass slide placed inside a Petri dish. PDMS mixed and degassed was poured on top of the sticker molds. Then, we degassed the set again and curated it on a hotplate at 95°C for approximately 30 min. We removed the flow chamber from the mold and punched two 1.5 mm holes at the extremities of the channel to serve as inlets using a ϕ1.5 mm biopsy punch (Kai group, BF-15F).

To bond the microwell array and flow chamber, we used PDMS-toluene glue (Chueh et al., 2007). To make the glue, we added the PDMS mix to equal parts of toluene 99% (Nacalai Tesque, 34122-15, LOT: V5K6223). We spin-coated the low-viscosity glue on a glass slide with a maximum rotational frequency of 3000 rpm for 30 s. Subsequently, the bottom of the flow chamber was stamped on a glass slide coated with PDMS-toluene glue and the flow chamber was placed on the top of the microwell array. Finally, we placed the assembled device on a hotplate at 95°C for 30 min to cure the glue. A diagram of the procedure is shown in Supplementary Figure 1A and the assembled device is shown in Supplementary Figure 1B.

### Confocal Microscope Observation

We used a fluorescence microscope IX81 (Olympus Corporation) equipped with a spinning-disk confocal system (Yokogawa CSU-X1) (488 nm and 561 nm lasers, Coherent Obis) and an EM CCD camera (Andor and iXon X3). We collected the images with software Andor iQ v.3.6.2, and analyzed them using ImageJ software (Bourne and Bourne, 2010), which were uniformly corrected for brightness and contrast. For measuring particle size, we analyzed an area of 1.8 mm × 1.8 mm per sample per device with a resolution of 3.5 μm × 3.5 μm per pixel. We applied a uniform threshold to identify the particles and the dextran droplets and measured their areas with the *Analyze particles* of ImageJ, excluding particles smaller than 1 μm^2^, to prevent background noise from being counted as particles. From this area, we calculated the approximate radius of dextran droplets, assuming they were shaped like the microwell, and of DNA hydrogel particles, assuming they were spherical. The average number of particles per microwell was obtained by dividing the number of DNA hydrogel particles in an image by the number of dextran droplets.

## Results

**FIGURE 2:**
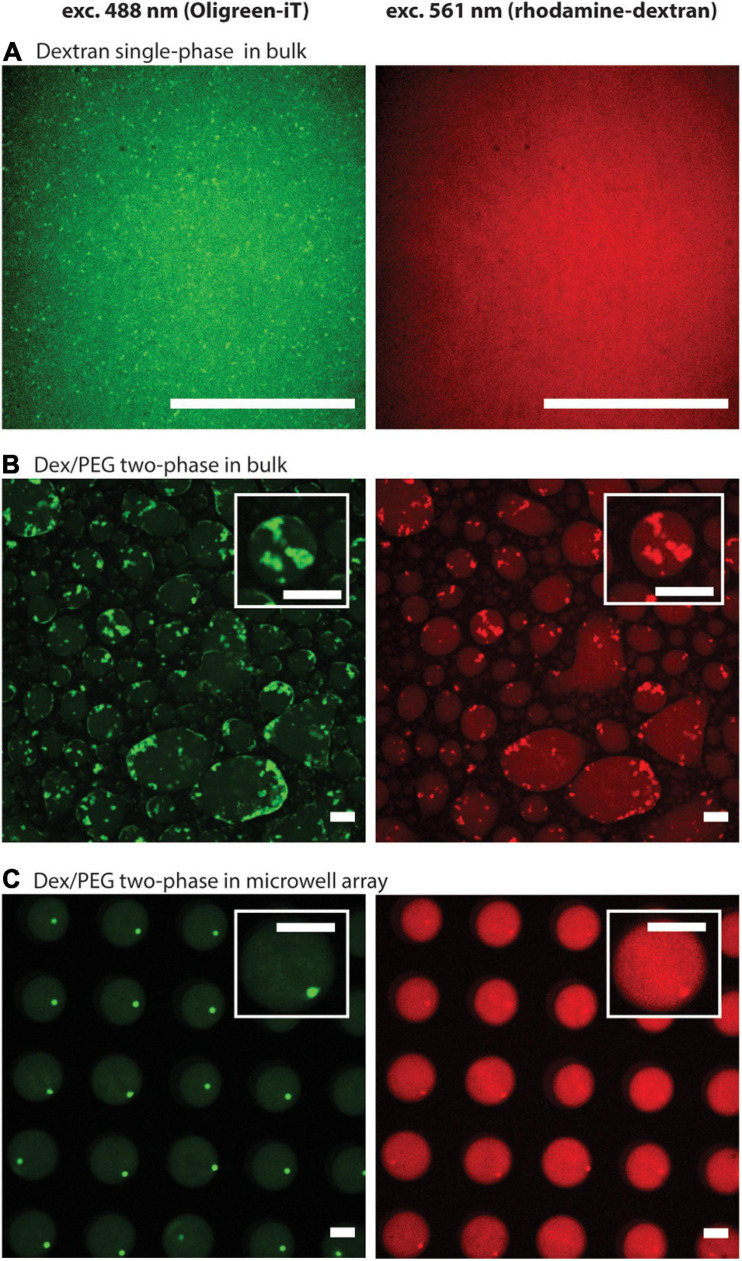
Formation of DNA hydrogel particles in single/two-phase systems and in bulk solutions/microwell arrays after two annealing rounds, displaying the location of the DNA aggregates (left column), and the dextran phase (right column). **(A)** Dextran single-phase system in a bulk solution: contains 8.33% w/w dextran, 8 μM per strand of DNA, 1.25 μM spermine, and 2.5 mM magnesium ions. **(B)** Dex/PEG two-phase system in a bulk solution: 5 μL containing 8.33 % w/w dextran, 8 μM per strand of DNA, mixed with 15 μL containing 8.33% w/w PEG, 1.66 μM spermine, and 3.33 mM magnesium ions. **(C)** Dex/PEG two-phase in a microwell array: array filled 8.33% w/w dextran, 8 μM per strand of DNA, and washed with a solution containing 8.33% w/w PEG, 1.25 μM spermine, and 2.5 mM magnesium ions. In all samples the dextran phase contains 10 mM Tris–HCl, 0.01% w/w rhodamine-dextran, 2/10000-diluted Oligreen. The PEG phase contains 10 mM Tris–HCl. About the conditions, see also Supplementary Table 2. Scale bars = 50 μm.

First, we compared the formation of DNA hydrogels using 8 μM of each DNA strand (Y1, Y2, and Y3) in the dextran single-phase system in a bulk solution, the Dex/PEG two-phase system in a bulk solution, and the Dex/PEG two-phase system in the microdroplet array. We observed the localization of DNA via the fluorescence of Oligreen (Figure 2, left column) and the localization of the dextran phase via fluorescence of the rhodamine-dextran probe (Figure 2, right column). In the single-phase dextran in the bulk solution (Figure 2A), small DNA hydrogel particles were formed with a strong background signal, indicating that a large fraction of the DNA was dispersed rather than being in the gel form. In the Dex/PEG two-phase system in the bulk solution, large, polydisperse DNA hydrogel particles were formed in the Dex/PEG droplets (Figure 2B). The DNA hydrogel particles were concentrated inside the dextran-rich droplets. We also observed that the position of the particles coincided with a higher fluorescence of the dextran probe; it was not clear whether this happened owing to cross fluorescence or whether the fluorescent probe had an affinity for the DNA aggregate. However, we observed that the absence of the dextran-rhodamine probe did not affect the formation of DNA hydrogel at 1 μM concentration (Supplementary Figure 2). In the Dex/PEG two-phase system in the microwell array, the Dex/PEG droplets were hosted inside the microwells of a microwell array and isolated from each other by a PEG-rich phase (Figure 2C). We observed that these dextran droplets were stable and uniform, and that single monodisperse DNA hydrogel particles were formed in the dextran droplets and tended to accumulate at the interface between the dextran and PEG phases.

**FIGURE 3:**
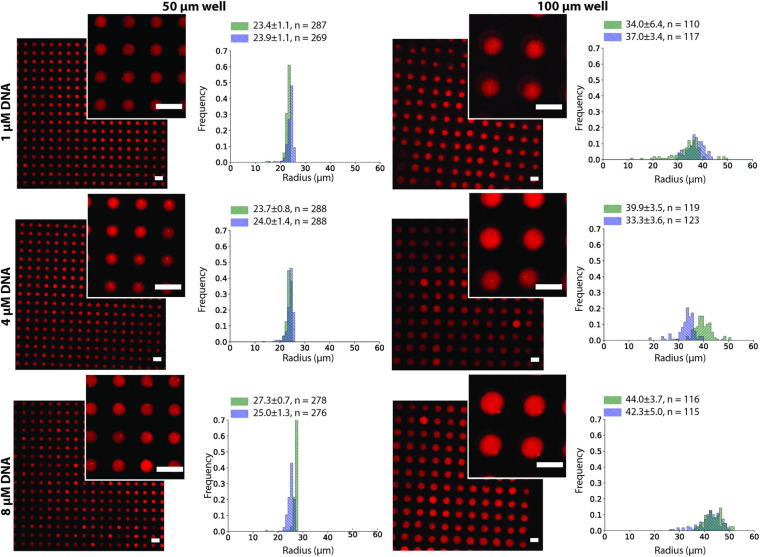
Dex/PEG microdroplet array stained by rhodamine-dextran in 50- and 100-μm microwells, displaying the droplet size distribution at different DNA concentrations for two separate experiments after two annealing rounds. Labels indicate the average, standard deviation, and size of each sample. Scale bars = 100 μm.

To test the effect of the microwell size and DNA concentration on the stability of the ATPS in the microwell array, we conducted the same experiment as in Figure 2C using 1, 4, and 8 μM of each DNA strand in the dextran solution and changed the microwell size to 50 or 100 μm (Figure 3). The stability was evaluated by comparing the size of the well with the size distribution of the droplets. Droplets smaller than the well size indicated that part of the droplet was washed away and were considered less stable. Although 200 μm microwells were also fabricated, they were unable to produce uniform-sized droplets, as most of the dextran was washed away during the insertion of the PEG phase (Supplementary Figure 3). Microwells sized 50 μm and 100 μm were able to produce uniform dextran droplets. The 50 μm microwells had a narrow droplet size distribution, and their size closely followed the size of the microwells. For the 100 μm microwells, however, the droplets were smaller than the microwell size, with an average of 70-90 μm, depending on the experiments. The DNA concentration had no obvious impact on the stability of the dextran droplets.

**FIGURE 4:**
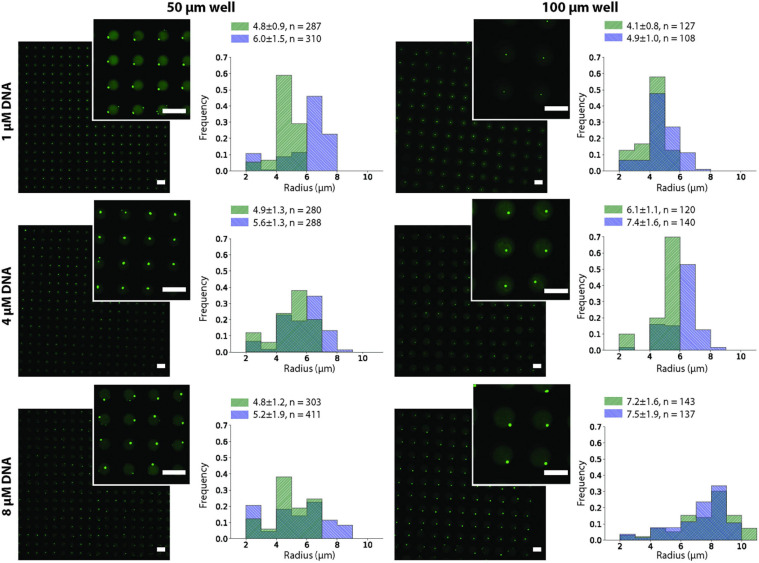
DNA hydrogel particles in Dex/PEG microdroplet array stained by Oligreen in 50- and 100-μm microwells, displaying their size distribution at different DNA concentrations for two separate experiments after two annealing rounds. Labels indicate the average, standard deviation, and size of each sample. Scale bars = 100 μm.

We observed that after the two annealing rounds, individual particles formed inside each Dex/PEG droplet. We measured the effect of the DNA concentration and microwell size on the distribution of the DNA hydrogel particles (Figure 4). For the 50-μm microwells, the difference in the average size of the DNA hydrogel particles was insignificant, with an average radius of 4.8 − 6.0 μm even for various DNA concentrations. For the 100-μm microwells, however, higher DNA concentrations increased the average size of the particles. Although the size of the DNA hydrogel particles could be controlled by increasing the DNA concentration, the average number of particles per microwell increased when the DNA concentration was 8 μM (Table 1). Additional images of the DNA hydrogels in 50- and 100-μm microwells are shown in Supplementary Figure 4.

**TABLE 1:** Average and standard deviation of number of DNA hydrogel particles per well according to microwell size and DNA concentration.

		**DNA concentration**		
		**1 μM**	**4 μM**	**8 μM**
**Microwell size**	50 μm	1.045 ± 0.034	0.986 ± 0.013	1.288 ± 0.190
	100 μm	1.046 ± 0.038	1.073 ± 0.064	1.212 ± 0.019

**FIGURE 5:**
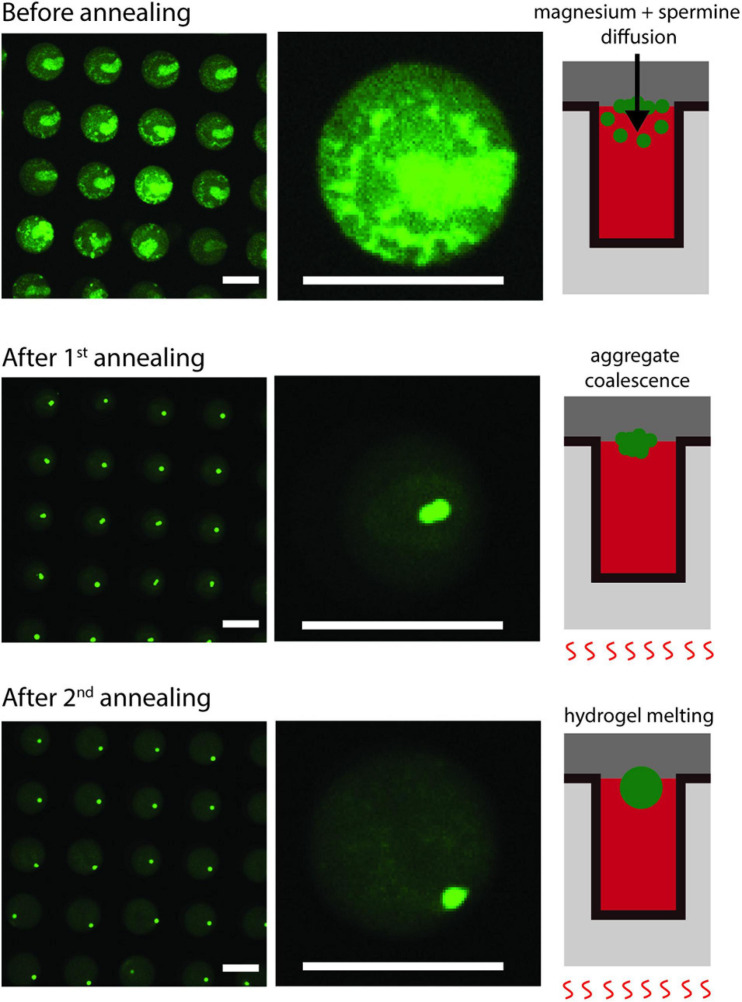
Location of DNA aggregates in Dex/PEG microdroplet array before and after each annealing round (left and middle column) with a schematic model of particle formation (right column). Scale bars = 100 μm.

During the formation of the hydrogels, they were twice subjected to annealing procedures, in which the temperature decayed exponentially, as measured with a thermal camera (Supplementary Figure 5) and considering a glass emissivity of 0.95 (Dvurechensky et al., 1979). To gain insight into the way in which the DNA hydrogel was formed, we monitored the 100-μm microwells before and after each annealing procedure (Figure 5). The probe Oligreen displays fluorescence of both single and double stranded DNA, with a higher fluorescence when bound to dsDNA, therefore the formation DNA hydrogel is followed by an increase in fluorescence. We observed that, after the flow of the PEG solution containing the magnesium ions and spermine, DNA aggregates formed on the interface of the Dex/PEG droplets. After the first annealing procedure, more compact particles formed, and the background fluorescence of Oligreen decreased, indicating that a larger fraction of the DNA was in the gel form instead of in solution. After the second annealing step, the aggregates were spherical and resided at the interface of the Dex/PEG droplets.

We noted that the presence of spermine was a determinant of the formation of the DNA hydrogel and its morphology. Samples that contained magnesium ions but no spermine were not able to form DNA hydrogel particles (Supplementary Figure 6A), whereas samples that contained 2.5 μM spermine (Supplementary Figure 6B) formed aggregates that did not melt during annealing, but retained the aggregated shape. For these samples, we observed a convective flow within the Dex/PEG droplet after the first annealing step. The convective flow facilitated the fusion of smaller aggregates into a larger aggregate at the top center of the Dex/PEG droplet (Supplementary Movies 1, 2).

## Discussion

In this study, we demonstrated the formation of single, uniform-sized DNA hydrogel particles in Dex/PEG droplets in a microwell array (Figure 2). The formation of stable Dex/PEG droplets depended on the microwell size (Figure 3 and Supplementary Figure 3), and the DNA hydrogel particle size could be controlled by changing the DNA concentration with the 100-μm microwell array (Figure 4). The process of formation was revealed; that is, aggregates accumulated at the dextran-PEG interface, and their coalescence was induced by successive annealing rounds of the device (Figure 5).

DNA hydrogel formation typically requires a high salt concentration (Nguyen and Saleh, 2017), which limits their application under physiological conditions. Therefore, for applications such as drug delivery or gene transfer, it is necessary to determine the conditions under which DNA hydrogel formation is enhanced at lower salt concentrations. We found that the Dex/PEG two-phase system could generate DNA hydrogels even at a low cation concentration (2.5 mM magnesium ions and 1.25 μM of spermine). These results suggest that formation methods based on ATPS could facilitate the identification of suitable conditions of DNA hydrogel formation at physiological conditions.

Figure 2 shows that only small DNA hydrogel particles were present in the dextran single-phase system, but large DNA hydrogel aggregates could form in the Dex/PEG two-phase systems in both the bulk solution and the microwell array. This comparison enabled us to conclude that the Dex/PEG two-phase system enhanced the formation of the DNA hydrogel. We elaborated the following two hypotheses to explain why ATPS contributes to the formation of larger DNA hydrogel particles.

The first hypothesis is that macromolecular crowding effects, such as a depletion force, are stronger in the Dex/PEG two-phase systems than in the dextran single-phase system and favor DNA aggregation. The depletion force is a force with an entropic origin often seen in macromolecular crowding environments that manifest in polymer solutions, favoring the self-assembly of biopolymers (Marenduzzo et al., 2006), and which can stabilize the DNA duplex (Nakano et al., 2004; Hong et al., 2020). The assembly of DNA tile microtubes (Zhang et al., 2020) and compacting genomic DNA (Zhang et al., 2009) under macromolecular crowding conditions have also been reported. In Figure 2, the dextran concentration in the dextran phase in both the single- and two-phase systems was the same; however, the dextran droplets in the two-phase systems contained a small amount of PEG molecules. Even if the PEG concentration inside the dextran droplets was low, the overall macromolecular crowding effects in the Dex/PEG two-phase systems would be higher than those in the dextran single-phase system.

The second hypothesis is related to the inhomogeneity of ion concentration in the solution. Johansson reported that various ions often display a preferred phase in ATPSs (Johansson, 1970). The preferential partition of ions was shown to create an electric potential across the dextran and PEG two-phase systems. The electric potential affects the partitioning of positively and negatively charged polyelectrolytes (Albertsson, 1971). This phenomenon is known as the Donnan effect, and it can be used to increase or decrease the concentration of charged polymers in each of the two phases by the addition of specific salts (Albertsson, 1971). Therefore, if magnesium acetate or spermine display a preferential partition, they could enhance the accumulation of DNA inside dextran droplets, resulting in the enhancement of the formation of the DNA hydrogel.

We observed that the size of the DNA hydrogel particles could be partially controlled by the DNA concentration, although the effect was more pronounced in the 100-μm microwells than in the 50-μm microwells. We constructed a simple model and compared it to the observed size.

**FIGURE 6:**
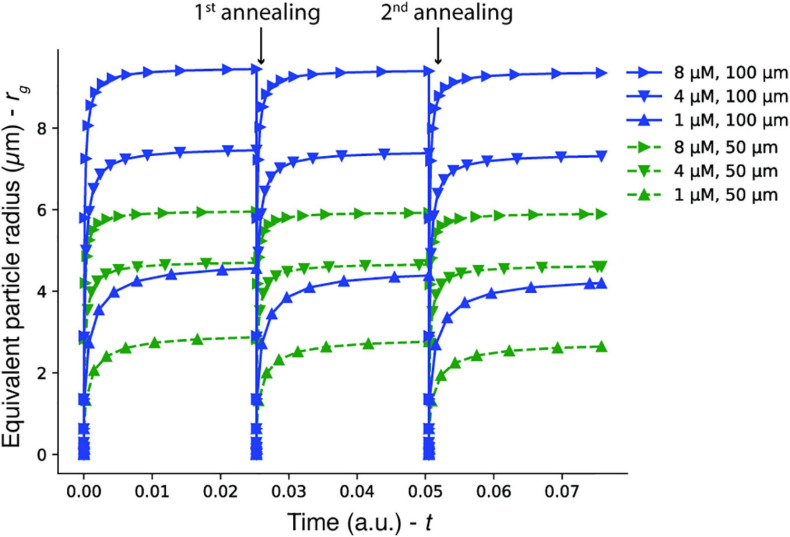
Model of DNA aggregate growth according to initial DNA concentration and microwell size.

In this model, we consider that unbound Y-motifs can diffuse out of the Dex/PEG droplet in a microwell or bind to other Y-motifs and that the binding is irreversible for simplicity. In the bound form, the Y-motifs do not diffuse out of the dextran phase. The interface of the Dex/PEG droplet has a surface area *A*, and the microwell has a height *h*, such that the droplet volume is approximately *V*=*Ah*. The concentration of Y-motifs was *c_u*, and the absolute number of unbound Y-motifs in the Dex/PEG droplet was *N*_*u*_=*c*_*u*_*V*. We assume that *c_u* is uniform along the microwell and that Y-motifs do not exist outside the microwell at all times. The concentration gradient around the dextran-PEG interface can be assumed to be *c*_*u*_/*w*=*N*_*u*_/(*Vw*), where *w* is the thickness of the dextran-PEG interface if it is modeled as a semi-permeable membrane. The unbound Y-motifs then diffuse out of the dextran droplet with a flux given by Fick’s law: −*Dc*_*u*_/*w* = −*DN*_*u*_/(*Vw*), where *D* is the diffusion coefficient of the unbound Y-motifs. Given that the Y-motifs are monodisperse spheres with radius *r*, the diffusion coefficient is *D*=*kT*/(6πη*r*), where *k*,*T*,η are the Boltzmann constant, temperature, and dextran viscosity, respectively. At the same time, the Y-motifs coalesce with each other to form aggregates, which we consider not to diffuse out of the microwell. The rate of the Y-motifs that coalesce is given by the Smoluchoswki coalescence equation for monodisperse particles: $\text{d}\,c_{u}/\text{d}\,t=-\left(\alpha /2\right)\,c_{u}^{2}$, where α is the rate of particle collisions, that is, (1/*V*)d*N*_*u*_/d*t* = −(α/2)(*N*_*u*_/*V*)^2^. For spheres that collide owing to Brownian motion, α=8*kT*/(3η) (Hunt, 1980). Therefore, the rate of change of the Y-motifs owing to diffusion and aggregation is:

$$\frac{\text{d}\,N_{u}}{\text{d}\,t}=-\frac{k\,T}{6\,\pi \,\eta \,r}\,\frac{A}{V\,w}\,N_{u}-\frac{4\,k\,T}{3\,\eta }\,\frac{1}{V}\,N_{u}^{2}$$

where, *N*_*u*_(0) is the initial condition of the unbound Y-motif. Given that all Y-motifs that form dimers further coalesce into a single aggregate, the rate at which the number of Y-motifs in the aggregate increase is assumed to be

$$\frac{\text{d}\,N_{g}}{\text{d}\,t}=\frac{4\,k\,T}{3\,\eta }\,\frac{1}{V}\,N_{u}^{2}$$

with *N*_*g*_(0)=0. We assume that during each annealing round, the aggregates return to their dissociated state. Given that ρ_*g*_is the molar concentration of the Y-motif in the hydrogel, $\left(4\,\pi \,r_{g}^{3}/3\right)\,\rho_{g}=N_{g}$. Therefore, we have:

$$r_{g}=\frac{1}{\lambda }\,{\left(\frac{N_{g}}{\rho_{g}}\,\frac{3}{4\,\pi }\right)}^{1/3}$$

where, λ is the compaction factor of DNA, which is expected because of the addition of crowding agents and spermine (Raspaud et al., 1999). To estimate *r_g* for each microwell size and DNA concentration, we considered *T*=298K,η=10^−2^*Pa*⋅s (based on a linear extrapolation of existing measurements (Neu et al., 2008) for dextran 250 kDa), *r* = 5 (given the length of each stem of the Y-motif), *d*=40nm [based on an approximation of the length of the interfacial region of gelatin-dextran ATPS (Vis et al., 2015)], ρ_*G*_ = 14 μM [measured by Sato et al. (2020)], and λ=5 [based on the simulation of the change in the radius of a flexible polymer in the presence of crowding agents (Kim et al., 2015)], with *A*,*V*,*Y*_*u*_(0) calculated for each microwell size and DNA concentration. Solving the equations for these parameters, we obtain the results in Figure 6. These results suggest that the dependence of the size of the DNA hydrogel particle on the initial DNA concentration for 100-μm microwells is more significant than that for 50-μm microwells. The qualitative tendency agrees with the experimental results shown in Figure 4.

In this model, the ATPS was represented as a semi-permeable membrane that allowed the flow of monomers, but not of aggregates larger than dimers. The goal of this model was to demonstrate that the compartmentalization of DNA in ATPS is more complex than that in W/O emulsions and that diffusion affects the size of particles assembled inside it. This leads to the question of whether ATPS can be used as a cell model. Jia et al. (2014) observed that for short RNA sequences, molecules are rapidly exchanged between phases, which is not desirable when using ATPS as a model of a prebiotic cell. However, Tsumoto et al. (2015) reported that the partition of nucleic acids depends on their size, in which case one could argue that ATPSs are more similar to physiological membranes than liposomes because of their semi-permeable and size-selective nature. Furthermore, it is possible to coat ATPS phases by lipid structures (Long et al., 2005; Sakuta et al., 2020; Zhang et al., 2021), which might allow modulation of the exchange of molecules between phases.

The localization of the aggregates at the interface seems to contribute to the formation of a single particle because it limits their position to the 2D interface instead of being distributed throughout the microwell and allows it to coalesce at once during the annealing rounds. The reason for DNA accumulation at the interface could be the limited diffusion of the DNA hydrogel. The accumulation at the surface occurs as a result of surface interactions between the DNA hydrogel particles and the dextran and PEG phases. According to Albertsson (1971), given that γ_p,*PEG*_ is the interfacial tension between the hydrogel particle and the PEG phase, γ_p,*dex*_ is the interfacial tension between the DNA hydrogel particle and dextran, γ_*dex*,*PEG*_ is the interfacial tension between dextran and PEG, and the accumulation of a particle at the interface occurs when the following condition is satisfied: |*γ*_p,*PEG*_−*γ*_p,*dex*_| < *γ*_*dex*,*PEG*_. The value γ_*dex*,*PEG*_, for example, can be adjusted by the concentration of PEG and dextran (Liu et al., 2012); therefore, to promote particle accumulation at the interface, a higher concentration of dextran and PEG should be used. An additional reason for the accumulation of DNA aggregates at the Dex/PEG interface is the phenomenon of depletion forces near soft surfaces (Bickel, 2003). The phase separation observed in the Dex/PEG droplet is visually analogous to that observed for DNA/PEG (Biswas et al., 2012) and DNA/alginate droplets in W/O emulsions (Negishi et al., 2011). In this type of phase separation, a colloidal suspension accumulates near a deformable surface owing to the depletion force caused, for example, by polymers in the solution. However, it is not clear whether the Dex/PEG interface can be modeled as a soft wall, and the phenomenon of the appearance of depletion forces at an aqueous-aqueous interface remains to be studied.

The last aspect that could contribute to the formation of single particles inside the microwells is the convective flow within the droplet. When a higher concentration of 2.5 μM spermine was used, we observed that multiple aggregates were formed after the initial annealing round. These aggregates were joined when aided by the flow within the droplet (Supplementary Movies 1, 2). We believe this flow might have been caused by the temperature difference between the top and bottom of the device during the first annealing round, which caused thermocapillary convection in the two-phase system (Liu et al., 1998). We expect that future experiments would enable the mechanism for the formation of single particles in the Dex/PEG droplet to be clarified further.

## Conclusion

Aqueous two-phase systems enable the formation of large DNA hydrogel particles at low magnesium ion concentrations, which has favorable implications for their use under physiological conditions and might provide insight into the stability of DNA coacervates and self-assembled structures. When the ATPS was formed inside a microwell array, the emulsion in the microwells was stable. When the ternary mixture of DNA/dextran/PEG was annealed in a microdroplet array, an aqueous triple-phase system was formed. The droplet array yielded a single large DNA hydrogel particle per microwell, the size range of which could be controlled by selecting the microwell size and DNA concentration. We expect it to be possible to use the same technique to generate monodisperse particles of other gels that undergo thermal gelation and that the DNA/Dex/PEG systems could be further developed as a reproducible and controllable model for artificial cells and microchemical reactors.

## Data Availability Statement

The raw data supporting the conclusions of this article will be made available by the authors, without undue reservation.

## Author Contributions

MM performed the experiments. MM and YO fabricated the microfluidic devices. MM and MT wrote the manuscript and conceived the original idea. MT supervised the project. All authors contributed to the article and approved the submitted version.

## Conflict of Interest

The authors declare that the research was conducted in the absence of any commercial or financial relationships that could be construed as a potential conflict of interest.

## Publisher’s Note

All claims expressed in this article are solely those of the authors and do not necessarily represent those of their affiliated organizations, or those of the publisher, the editors and the reviewers. Any product that may be evaluated in this article, or claim that may be made by its manufacturer, is not guaranteed or endorsed by the publisher.

## Footnotes

1 Along the text, we will refer to dextran-in-PEG (Dex/PEG) emulsions as those in which dextran constitutes the droplet phase and PEG constitutes the continuous phase.
