# Supplementary material for: Aqueous Triple-Phase System in Microwell Array for Generating Uniform-Sized DNA Hydrogel Particles
Source: Front Genet. 2021 Jul 23;12:705022. doi: 10.3389/fgene.2021.705022 (PMC8343185; doi:10.3389/fgene.2021.705022)
Supplement: Supplementary file 3 [file Presentation_1.pdf]

Supplementary Material

1 Supplementary Tables

Supplementary Table 1 – Composition of solutions used in the experiments.

	(i)	(ii)	(iii)	(iv)
Tirs-HCl	10 mM	10 mM	10 mM	10 mM
Dextran	8.33 % w/w	8.33 % w/w	-	-
Rhodamine-dextran	0.01% w/w	0.01% w/w	-	-
Oligreen	2/1000	2/1000	-	-
PEG	-	-	8.33% w/w	8.33% w/w
Magnesium acetate	2.5 mM	-	3.33 mM	2.5 mM
Spermine	1.25 µM	-	1.66 µM	1.25 µM
DNA (Y1, Y2, Y3)	8 µM/each	8 µM/each	-	-

Supplementary Table 2 – Experimental conditions.

	Dextran single-phase system in a bulk solution	Dex/PEG two-phase system in a bulk solution	Dex/PEG two-phase system in a microwell array
Mixing solutions	20 µL of solution (i)	5 µL of solution (ii)<br>+ 15 µL of solution (iii)	Solution (ii) inserted in microwells and washed with solution (iv)
Final concentrations for experiments	10 mM Tris-HCl<br><br>8.33% w/w dextran<br><br>0.01% w/w rhodamine-dextran<br><br>2/10000 Oligreen for DNA staining<br><br>2.5 mM magnesium acetate<br><br>1.25 µM spermine<br><br>8 µM/each Y1, Y2, Y3<br><br>Without PEG	10 mM Tris-HCl<br><br>~8.33% w/w dextran in droplet<br><br>~0.01% w/w rhodamine-dextran in droplet<br><br>2/10000 Oligreen for initial DNA staining<br><br>2.5 mM magnesium acetate<br><br>1.25 µM spermine<br><br>~8 µM/each Y1, Y2, Y3<br><br>~8.33% w/w PEG as continuous phase	10 mM Tris-HCl<br><br>~8.33% w/w dextran in droplet<br><br>~0.01% w/w rhodamine-dextran in droplet<br><br>2/10000 Oligreen for initial DNA staining<br><br>~2.5 mM magnesium acetate<br><br>~1.25 µM spermine<br><br>~8 µM/each Y1, Y2, Y3<br><br>~8.33% w/w PEG as continuous phase in flow channel

2 Supplementary Figures

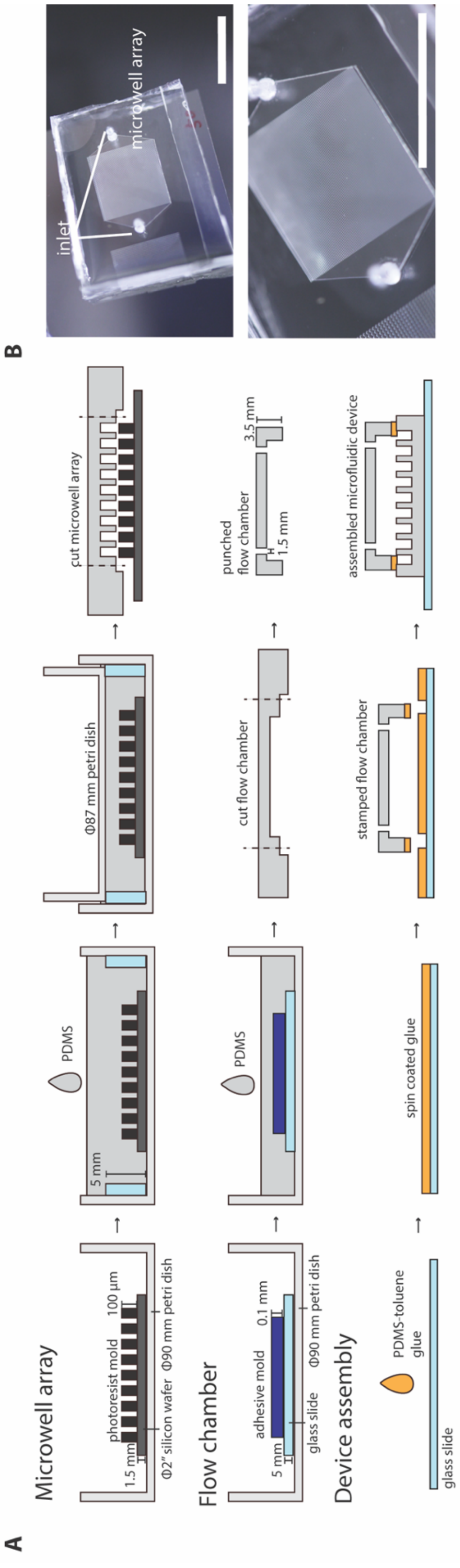

Supplementary Figure 1. A. Fabrication method of the microwell array microfluidic device. The microwell array was made in three steps: fabrication of PDMS microwell array, fabrication of PDMS flow chamber, and their assembly. The microwell array was based on a photoresist mold sandwiched between Petri dishes, whose height was controlled by a glass spacer. The flow chamber was based on a sticker mold and a typical casting procedure. The microwell array and the flow chamber were joined using a PDMS-toluene glue. **B.** Assembled microfluidic device. Scale bars: 1 cm.

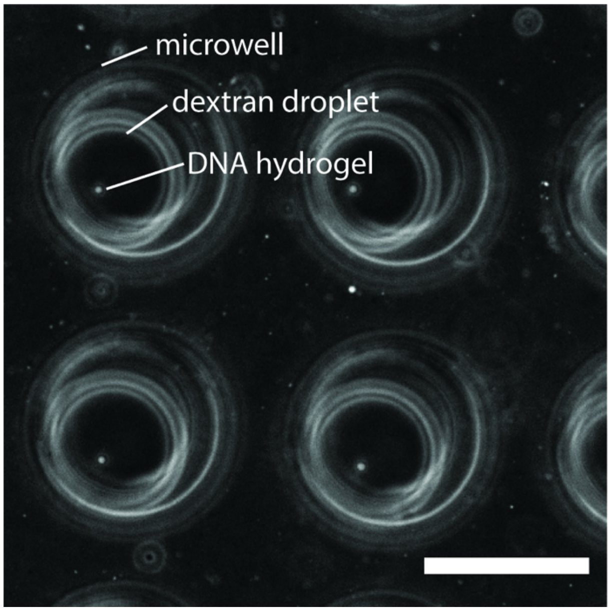

Supplementary Figure 2. DNA hydrogel particle in dextran droplet microarray observed by brightfield. The dextran phase contains 8.33% w/w dextran, 10 mM Tris-HCl, 1 µM Y1, Y2, Y3 DNA. The PEG phase contains 8.33% w/w PEG, 1.25 µM spermine, 2.5 mM magnesium acetate, 10 mM Tris-HCl.

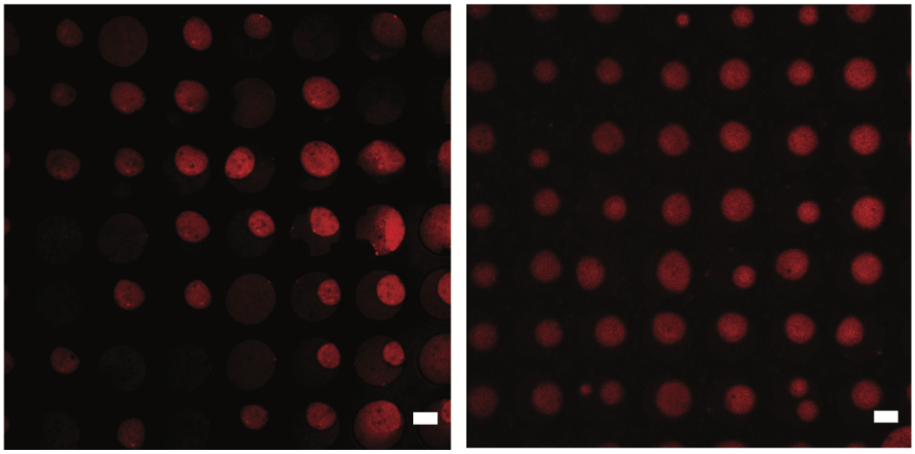

Supplementary Figure 3. Dextran droplets are unstable in microwells sized 200 µm. The dextran phase contains 8.33% w/w dextran, 10 mM Tris-HCl, 0.01% w/w rhodamine-dextran. The PEG phase contains 8.33% w/w PEG, 1.25 µM spermine, 2.5 mM magnesium acetate, 10 mM Tris-HCl. Scale bars: 100 µm.

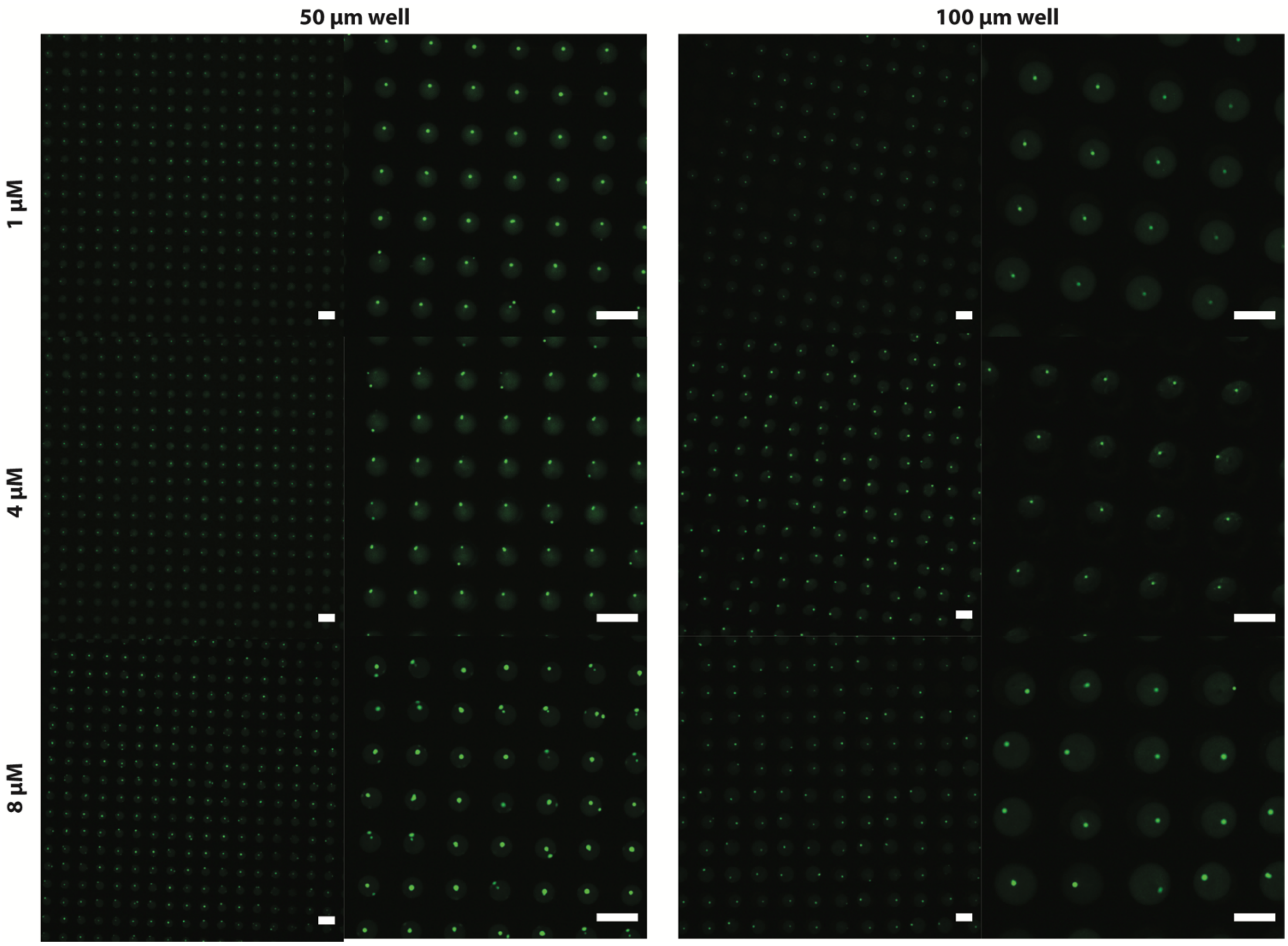

Supplementary Figure 4. Additional images of DNA hydrogel particles in microwells with 50 and 100 µm, containing DNA in concentrations of 1, 4, or 8 µM per strand. In all samples the dextran phase contains 10 mM Tris-HCl, 8.33% w/w dextran, 0.01% w/w rhodamine-dextran, 2/10000 diluted Oligreen. The PEG phase contains 10 mM Tris-HCl, 8.33% w/w PEG, 1.25 µM spermine, 2.5 mM magnesium acetate. Scale bars: 100 µm.

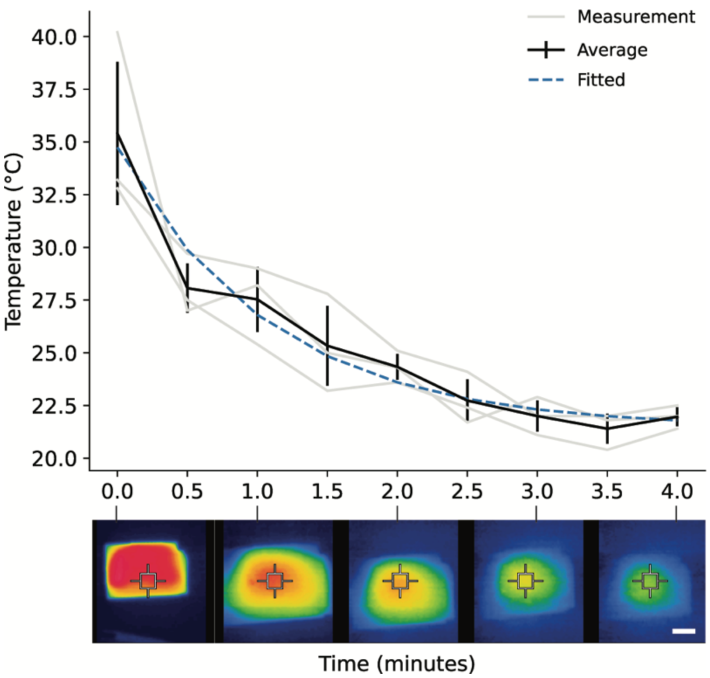

Supplementary Figure 5. Temperature of the bottom of the microfluidic device after heating on the hotplate for 1 minute at 75°C. The temperature was measured at the center of the device with a thermal camera FLIR TG167 at 10 cm away from the device, considering the glass has an emissivity of 0.95. Three separate measurements were done, averaged and an exponential decay $T(t) = Ae^{-\lambda t} + B$ was fit to the average, yielding constants of A = 10.43, $\lambda = 0.63$, B = 24.73. The bottom inset shows a sample of thermal images captured at each time. Scale bar: 1 cm.

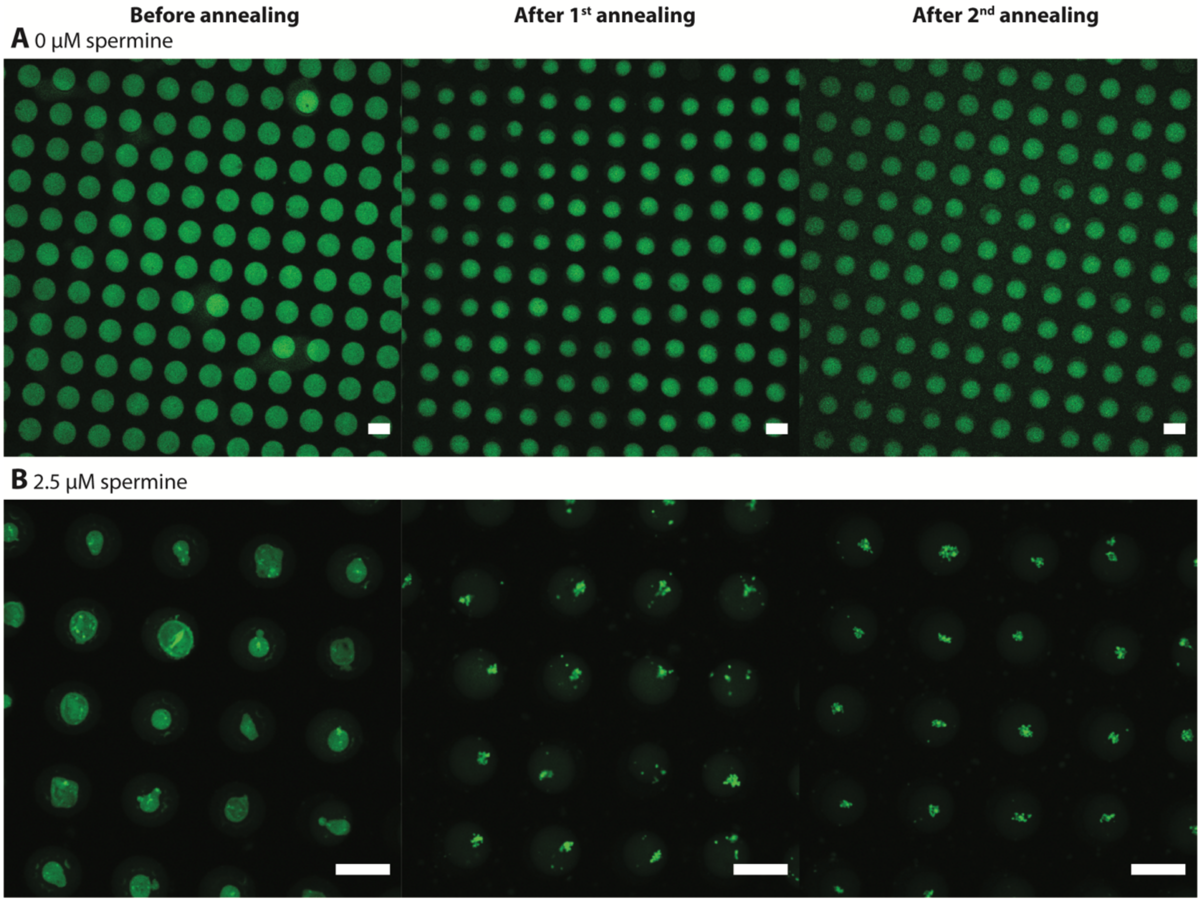

Supplementary Figure 6. Effect of spermine on the formation of DNA hydrogel. Fluorescence displays the localization of DNA before annealing, after first annealing, and after second annealing for samples containing either 0 μM and (top row) and 2.5 μM of spermine in the PEG phase (bottom row). In both samples, the dextran phase contains 10 mM Tris-HCl, 8.33% w/w dextran, 8 μM each of Y1, Y2, Y3 strands, 0.01% w/w rhodamine-dextran, 2/10000 diluted Oligreen. Both PEG phase contains 10 mM Tris-HCl, 8.33% w/w PEG, 2.5 mM magnesium acetate.

Supplementary Movie 1. Flow inside Dex/PEG droplets after the first annealing round. Real-time.

Supplementary Movie 2. Flow inside Dex/PEG droplets after the first annealing round facilitates the assembly of larger particles by aggregation. Real-time.
